# Elucidation of transformation pathway of ketoprofen, ibuprofen, and furosemide in surface water and their occurrence in the aqueous environment using UHPLC-QTOF-MS

**DOI:** 10.1007/s00216-014-7614-1

**Published:** 2014-01-23

**Authors:** A. Jakimska, M. Śliwka-Kaszyńska, J. Reszczyńska, J. Namieśnik, A. Kot-Wasik

**Affiliations:** 1 Department of Analytical Chemistry, Chemical Faculty, Gdańsk University of Technology (GUT), G. Narutowicza Street 11/12, 80-233 Gdańsk, Poland; 2 Department of Organic Chemistry, Chemical Faculty, Gdańsk University of Technology (GUT), G. Narutowicza Street 11/12, 80-233 Gdańsk, Poland; 3 Department of Chemical Technology, Chemical Faculty, Gdańsk University of Technology (GUT), G. Narutowicza Street 11/12, 80-233 Gdańsk, Poland

**Keywords:** Pharmaceuticals, Irradiation, Aqueous environment, Transformation products, Transformation pathway

## Abstract

The identification and determination of transformation products (TPs) of pharmaceuticals is essential nowadays, in order to track their fate in the aqueous environment and, thus, to estimate the actual pollution. However, this is a challenging task due to the necessity to apply high-resolution instruments enable to detect known and unknown compounds. This work presents the use of liquid chromatography quadrupole time-of-flight mass spectrometry (LC-QTOF-MS) as a powerful tool for the identification of three selected pharmaceuticals, furosemide (FUR), ibuprofen (IBP), and ketoprofen (KET), and their TPs in various water samples. Laboratory degradation experiments were performed using xenon lamp as a source of the irradiation in order to simulate phototransformation processes which may occur in the environment. Furthermore, the photodegradation kinetics of three selected compounds were assessed in a reactor equipped with xenon lamp in river water samples. Five TPs of IBP, seven of KET, and five of FUR were identified; some of them are presented here for the first time. Accurate mass measurements and fragmentation pattern obtained during an LC-QTOF-MS analysis allowed for structure elucidation of TPs followed by the creation of transformation pathway of selected pharmaceuticals. Finally, different water samples (wastewater influent and effluent, river water, untreated and treated water) were analyzed in order to estimate the presence of parent and transformed compounds. Only KET was detected in untransformed form in considered samples. Most of the TPs of selected drugs were found at least once in all water samples. Although IBP and FUR were not present in water samples as parent compounds, their different TPs occur. A great potential of LC-QTOF-MS in the identification and structural elucidation of TPs in the environment, allowing the recognition of the fate of pharmaceuticals in the environment through the determination of transformation pathway, has been presented.

**Electronic supplementary material:**

The online version of this article (doi:10.1007/s00216-014-7614-1) contains supplementary material, which is available to authorized users.

**Figure:**
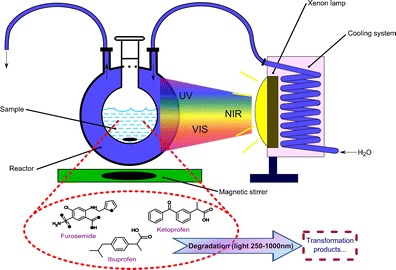
Phototransformation of three selected pharmaceuticals

## Introduction

Pharmaceutically active compounds (PhACs) have become an increasingly serious problem in recent years due to their continuous introduction to environmental waters from wastewater treatment plants (WWTPs) [1, 2]. Global consumption of PhACs and their use in human and veterinary medicine are now at an extremely high level. Once xenobiotics reach WWTPs, the fate of PhACs in the aquatic system can be divided into four ways [3]:

1. After series of transformations compounds are mineralized and organic matter is converted into simple inorganic compounds;
2. A compound, depending on its properties, may be less prone to degrade. If it has the physicochemical properties which enable to bind to the solid phase (i.e., hydrophobicity, lipophilicity), it may be partly accumulated in the sludge. In consequence, sorption will affect the ability to eliminate the compound during the treatment processes.
3. The compound can be transformed into a more lipophilic derivative than the primary compound, which is present in wastewater effluent.
4. A compound, which is stable and polar, is not retained or degraded in WWTPs and thus reaches the aquatic environment.

The problem of the PhAC presence in the effluent is that these compounds are generally stable. As far as it is a desirable property in medicine, it is redundant during wastewater treatment due to the limited removal of PhACs during biodegradation processes in conventional WWTP. However, the environmental degradation of compounds, such as biodegradation, photodegradation, and hydrolysis, may occur and depends on their chemical structure as well as weather conditions. These processes substantially reduce the potential of the compound to induce pharmacological effect; however, the formed transformation products may exhibit a similar activity and be more stable than the parent compound [4]. The exact risks associated with accurate and chronic exposure to random combinations of low levels of PhACs are not yet evaluated, although it may cause adverse effects on aquatic organisms. Since the level of pharmaceuticals is not regulated by law and they are considered as threat to the environment [5], attention has been paid to the environmental fate of these compounds in order to improve their efficient removal from WWTPs and prevent from continuous introduction of PhACs to aqueous environment [6].

Disinfection of drinking water and wastewater can be performed via ozonation, chlorination, or UV irradiation. The last one is often employed in water treatment plants (WTPs) due to its effectiveness in removing viruses and bacteria [7]. In Poland, the application of UV irradiation is rather limited due to high cost of the investment and maintenance. Although ultraviolet light has an additional advantage of removing PhACs [8], it can contribute to creating photodegradation intermediates that are more persistent or toxic than the parent analyte [9]. Laboratory experiments applying sunlight or mercury-vapor lamps on PhACs have proved that diclofenac, naproxen, ibuprofen, ketoprofen, and atenolol are susceptible to photodegradation in water samples (pure water river water, wastewater) [9, 10]. However, the degradation rates vary between studies what can be attributed to susceptibility of the compound to photolysis, matrix, and the type of the irradiation source. The phototransformation is considered as a potentially significant process in the degradation of pharmaceuticals [10] and involves direct photolysis (a compound absorbs the light by itself) and indirect photochemical reactions of a compound with active molecule, e.g., ·OH, ^1^O_2_, ROO·, ^3^DOM*, and e^−^_aq_ (that are generated by photosensitizers such as dissolved organic matter) [11, 12]. Under constant UV irradiation, degradation reactions are typically first order.

The pharmaceuticals (ketoprofen, ibuprofen—nonsteroidal anti-inflammatory drugs; furosemide—diuretic) chosen for this study were compounds which differ in term of chemical structure and were found in wastewater and river water in high concentrations (up to micrograms per liter [13, 14]). Little information is available on transformation products (TPs) of these compounds because most of the available reports are focused rather on parent compounds. While the awareness grows, the need for elucidating environmental fate of PhACs increases as well. The photodegradation of ketoprofen, ibuprofen, and furosemide has been briefly studied, mostly in pure water, surface water, and wastewater [9, 15, 16]. However, studies on TPs should be more accurate and extended to assess proper identification and elucidation of the structure of TPs and finding the pathway of their transformation in order to monitor the presence of transformed analytes in environmental water samples since some of the TPs were proved to be toxic [6, 17, 18].

Identification of TPs is based on suspected and nontarget screening what requires instruments generally with high-resolution and accurate mass measurements. To meet the present expectations of identifying known–unknown compounds at low concentrations in complex matrices, the coupling of liquid chromatography to high resolution mass accuracy (LC-HRMS) has become a powerful tool [19]. Such instruments as LC-quadrupole time-of-flight mass spectrometry (LC-QTOF-MS) are able to perform MS/MS experiments for obtaining full scan product ion spectra with accurate masses of fragment ions and thus provide structural information of the compound [20, 21]. The structure of identified compound is verified by comparing changes in exact molecular mass and masses of fragment ions which origin from parent ions [22]. Providing a reliable data is very important due to avoiding false-positive findings, and thus, high-resolution mass spectrometers such as QTOF-MS are required.

In light of these concerns and lack of literature data presenting the importance of degradation as a probable transformation pathway of PhACs, the aim of this study was to investigate the degradation kinetics and identification of TPs of chosen pharmaceuticals by UV/VIS irradiation in natural water samples. In the present study, the photodegradation kinetics of ibuprofen, ketoprofen, and furosemide was assessed in a reactor equipped with xenon lamp in river water samples. Furthermore, the TPs of each compound were identified with the application of LC-QTOF-MS. To the best of our knowledge, this is the most extensive study of TPs of ibuprofen, ketoprofen, and furosemide including kinetics and structure elucidation followed by proposing photodegradation pathways for each compound. Five degradation products of ibuprofen (IBP), seven products of ketoprofen (KET), and five of furosemide (FUR) were identified together with their structures. Some of these compounds are reported here for the first time following with new transformation pathways. Further monitoring of identified compounds in different water samples was carried out. The application of LC-HRMS provided the necessary confirmation due to the mass accuracy and structural information obtained during the fragmentation experiment.

## Materials and methods

### Reagents

Analytical standards of IBP, KET, and FUR were purchased from Sigma-Aldrich (Poznań, Poland). LC-MS grade methanol (MeOH) was obtained from Sigma-Aldrich (Poznań, Poland). Formic acid was from Merck (Warsaw, Poland). Ultrapure water was obtained from an HLP5 system (Hydrolab, Poland). MeOH used for extraction were LC grade and were purchased from Merck (Warsaw, Poland). Individual stock solutions of each compound at concentration level of 1 g/L were prepared in methanol and were stored at −80 °C.

### The irradiation experiment

**Fig. 1:**
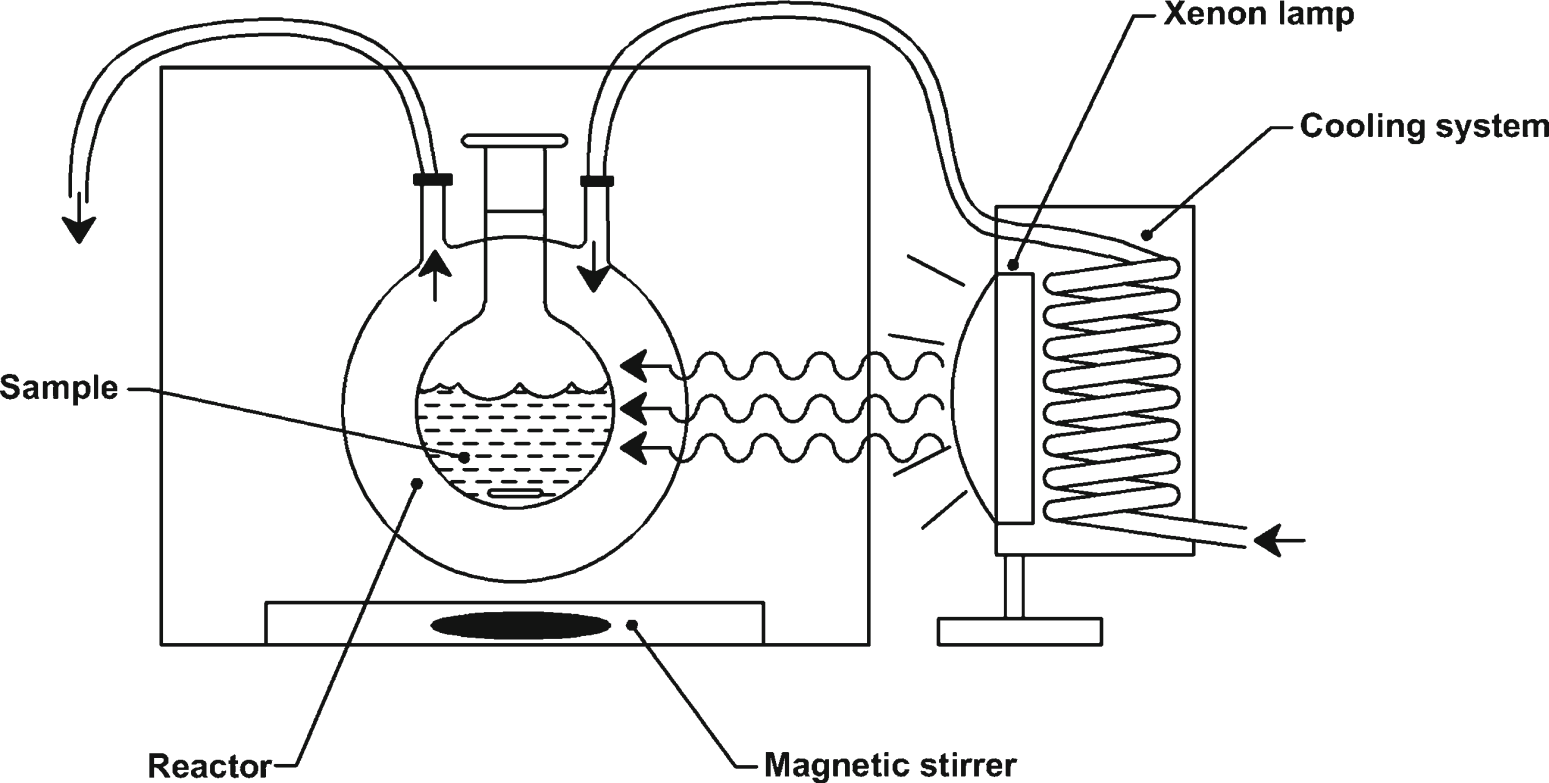
A small-scale system applied during the irradiation experiment

All degradation experiments were performed using a small-scale system that consisted of a cylindrical photoreactor (25 mL) equipped with quartz window and cooling system, external light system, and optical filters (Fig. 1). The irradiation was emitted by 1,000 W xenon lamp (627H, Oriel) and transmitted light of wavelength in range 250–1,000 nm due to the optical filters (UG1, Schott AG) used for cutting off the IR irradiation. Similar equipment was previously applied by Górska et al. [23] for photodegradation of phenol.

The temperature of the samples during the experiment was maintained at 10 °C. The photodegradation experiment was performed for each compound separately in river water free from the targets while providing the same experimental conditions. The initial concentration of chosen compounds (ibuprofen, ketoprofen, and furosemide) was 1 mg/L for both kinetic study and identification of TPs. Higher initial concentration of analytes increased the possibilities of proper identification and structure elucidation of TPs. Twenty-five milliliters of aqueous sample containing one of the compounds was stirred magnetically during the irradiation. Aliquots of about 500 μL were collected during the experiment at specific intervals dependent on the compound (total degradation time was different for each analyte), filtered through a 0.45-μm nylon syringe filter (Rockwood, USA), and subjected to ultra performance liquid chromatography (UPLC)-QTOF-MS analysis for further data evaluation.

### Analytical procedure

Analyses were performed on Agilent 1290 UPLC system (G4226A autosampler, G1312C binary pump and G1316A thermostated column). The separation of analytes was done using XTerra MS C18 (30 × 2.1 mm; 3.5 μm). The mobile phase A was ultrapure water with 0.05 % FA and B was MeOH at a flow rate 0.4 mL/min. The gradient started from 95 % A to 0 % A in 15 min, back to 95 % A in 1 min, and kept at 95 % A for 5 min. The column was maintained at 22 °C and the injection volume was 5 μL.

The UPLC system was connected to a hybrid QTOF mass spectrometer (Agilent 6540 Series Accurate Mass QTOF-MS) operated in negative ion mode. Ions were generated using a dual ESI ion source. Operation conditions were as follows: sheath gas temperature 400 °C at flow rate of 12 L/min, capillary voltage 4,000 V, nebulizer pressure 20 psig, drying gas 10 L/min, gas temperature 325 °C, skimmer voltage 45 V, octopole RF peak 750 V, and fragmentor voltage 100 V. Analyses were performed using MS/MS or AutoMS/MS mode with fixed collision energy and in mass range of 50–1,000 *m*/*z*. The instrument was operated in the 4-GHz high-resolution mode. Acquisition rate was 1.5 spectra/second. Acquisition data were processed with Agilent MassHunter Workstation software.

### Sample collection and preparation

Aqueous samples collected from the Gdańsk area where influent and effluent water samples were from a WWTP, treated and untreated water from a WTP, and river water from Radunia River exposed to anthropogenic activity. All samples were collected in prerinsed amber glass bottles and filled to the brim to reduce analyte transition to the gas phase. After immediate transportation to the laboratory, they were stored at 4 °C and analyzed within 24 h. Environmental water samples were screened for the presence of identified compounds.

Sample preparation was based on solid phase extraction (SPE) and was performed using Oasis HLB cartridges (200 mg, 6 mL; Waters). SPE cartridges were preconditioned with 5 mL of MeOH and 5 mL of ultrapure water. Before loading, 1 mL 0.1 M EDTA/100 mL sample was added. A volume of 100 and 200 mL of influent and effluent, respectively, and 500 mL of treated, untreated water, and river water were loaded on the sorbent at a flow rate of 5 mL/min (in case of extraordinary contaminated samples with solid particles, before loading, it was filtrated by a cellulose filter). The sorbent was dried for 15 min and then the analytes retained were eluted with 6 mL (3 × 2 mL) of MeOH. The extracts were evaporated to dryness in a stream of nitrogen and residues were redissolved in 1 mL of MeOH/H_2_O (1:9, *v*/*v*). Five microliters of this solution was injected into the UPLC-QTOF-MS system.

## Results and discussion

### Photodegradation kinetics in river water

**Fig. 2:**
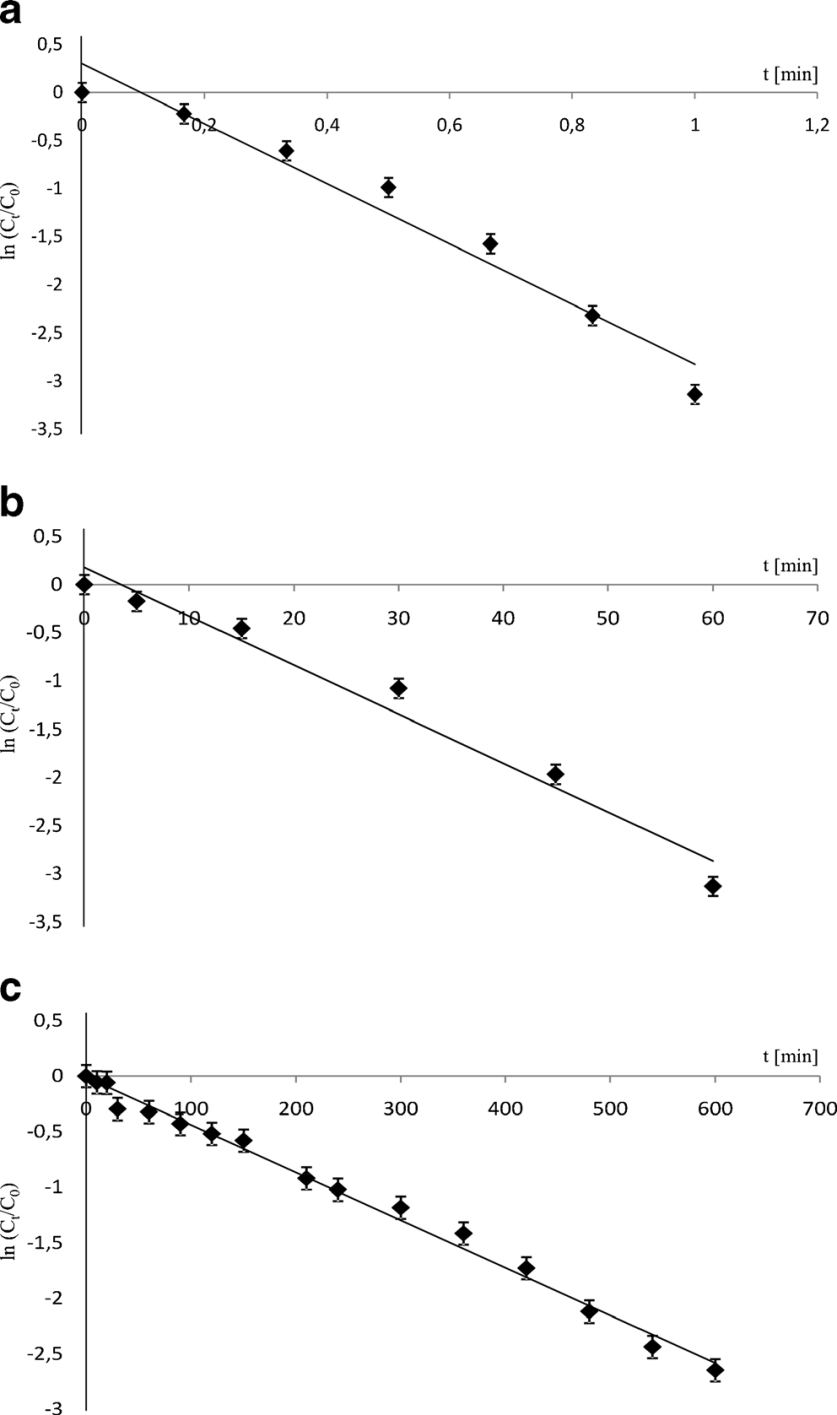
Degradation kinetics of **a** ketoprofen, **b** furosemide, and **c** ibuprofen in river water

The photodegradation of the three studied compounds in river water followed a pseudo-first-order kinetics (Fig. 2). The time-based pseudo-first-order rate constants (*k*) were determined according to the Eq. (1):

1 $$ ln\frac{C_t}{C_0}=- kt $$

where *C*_*t*_ is the concentration of the compound at the irradiation time *t* and *C*_0_ is the initial concentration of the analyte. The results were plotted using the natural logarithm of the analyte concentration as a function of time. Pseudo-first-order photodegradation rate constants were determined by regression analysis. Half-lives *t*_1/2_ were calculated using Eq. (2) derived by transforming Eq. (1) and replacing *C*_*t*_ with *C*_0_/2:

2 $$ {t}_{1/2}=\frac{ ln2}{k} $$

Photodegradation of selected compounds had different time-based degradation rate constants; the value of the constant *k* was one and three orders of magnitude higher for FUR and KET, respectively, than IBP (Table 1). After a different time of irradiation, analytes reached a final concentration level below detection limit. KET was undetectable after 60 s of exposition to Xe lamp light, FUR after 60 min, and IBP was the most persistent one degrading after 13 h of irradiation. Absorption spectra of KET (*λ*_max_ = 205 and 255) and FUR (*λ*_max_ = 228 and 275) overleap the Xe lamp emission spectrum which has an important influence on their degradation. On the other hand, the small absorbance in the UV region for IBP (*λ*_max_ = 222) helps to explain why its photodegradation was less efficient relatively to KET and FUR.

**Table 1:** Photodegradation rate constants (*k*), half-lives (*t*_1/2_), and irradiation time for IBP, KET, and FUR in river water

Compound	*k* (min^-1^)	*t*_1/2_ (min)	*t*_irradiation_ (min)
IBP	0.0039	180	780
KET	1.8	0.4	5
FUR	0.030	23	60

Overall, these parameters allow assuming that these compounds are probable to degrade at the wavelength range used and, as presented in this study, indicate a higher degradation potential for ketoprofen, followed by furosemide and then ibuprofen (Table 1 and Fig. 2).

### Phototransformation of selected pharmaceutical

In order to identify reaction products of the target compounds, samples collected at various time intervals were analyzed by LC-QTOF-MS. Exposure of the drug solutions in the river water to light allowed simulation of conditions naturally occurring in the environment. This was possible because Xe lamp emits continuous concentrated light which wavelength range overlaps the sunlight spectra (250–1,000 nm) that includes ultraviolet light, visible light, and near infrared light. Preliminary identification of the probable TPs was based on accurate mass measurements of the parent ion with a mass error <5 ppm in the full scan acquisition mode. To provide reliable confirmation of TPs during laboratory degradation experiment, further accurate MS/MS measurements were performed in order to observe characteristic fragmentation ions of the parent compound. Such approach is a safe way for an accurate identification and is helpful in the structure elucidation, especially in case the compounds are not present in a library.

The irradiation of the pharmaceuticals resulted in the formation of new chromatographic peaks corresponding to several TPs generated during the photodegradation processes. Five degradation products of IBP photolysis, seven products of KET, and five of FUR were identified and, in detail, are presented in Table 2. The TPs of the chosen pharmaceuticals were tentatively identified by extracted ion chromatograms using a 10-ppm error window while performing full scan analysis. As presented in Table 2, the experimental mass (*m*/*z*) deviation from the theoretical molecular masses for deprotonated [M-H]^−^ structural transformation compounds ranged from −5.4 to 5.1. The accurate mass MS/MS spectra of all compounds (Electronic Supplementary Material, Figs. S1, S2, and S3) were obtained by selecting the deprotonated molecule [M-H]^−^ at a specific *m*/*z* value as a precursor ion to yield the fragmentation pattern and thus to obtain the structural information of the compound. Accurate mass measurements with low mass error together with fragmentation pattern of TPs provided by LC-QTOF-MS analysis were the most important while elucidating structures and the transformation pathway of selected pharmaceuticals.

**Table 2:**
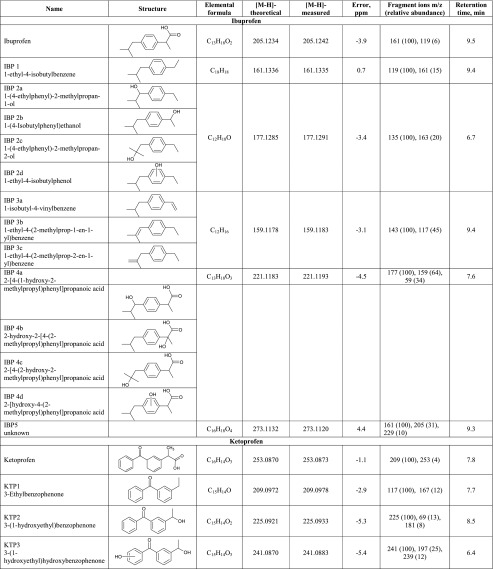

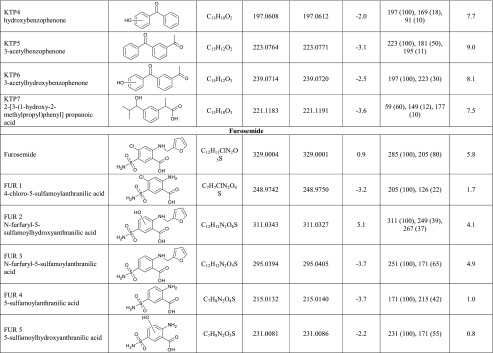
Identification of transformation products generated by Xe lamp photodegradation of IBP, KET, and FUR

#### Identification and confirmation of IBP degradation products through laboratory degradation experiment

The first product of degradation has been found immediately after preparation of its solution in river water, before exposure to light. The most important fragment in the mass spectrum of product IBP 1 is the ion of *m*/*z* 161.1335, which corresponds to decarboxylation of the parent molecule. Due to the fact that this product appeared quickly and without exposure to light, we assumed that its formation is probably caused by transformation processes, which may occur in river water (e.g., hydrolysis), rather than photolytic degradation. It must be noted that this degradation product (IBP 1) is already known in the literature as the product formed after photodegrative destruction as reported by Caviglioli et al. [24] for the chemical oxidation of this drug with permanganate, dichromate, and concentrated H_2_O_2_, by Skoumal et al. [25] for the electrochemical by electro-Fenton and photoelectro-Fenton degradation, by Szabo et al. [26] during the UV (254 nm) and vacuum ultraviolet UV/VUV (254/185 nm) photolysis, and by Mendez-Arriaga et al. [27] by heterogeneous TiO_2_ photocatalysis.

Exposition of the ibuprofen solution to Xe lamp light caused formation of the second degradation product (IBP 2) after 1.5 h. This product with the *m*/*z* value 177.1291 may correspond to mono-hydroxylated derivative of compound IBP 1. During the irradiation of river water with Xe lamp, hydroxyl radicals and other oxidants are formed according to the following reactions [28]:

$$ \begin{array}{l}{\mathrm{H}}_2\mathrm{O}+\mathrm{UV}\to \mathrm{H}\cdotp \kern0.5em +\kern0.5em  OH\mathit{\cdotp}\hfill \\ {} OH\mathit{\cdotp}\kern0.5em + OH\mathit{\cdotp}\kern0.5em \to {\mathrm{H}}_2{\mathrm{O}}_2\hfill \\ {}\mathrm{H}\cdotp \kern0.5em +\kern0.5em {\mathrm{O}}_2\to {\mathrm{H}\mathrm{O}}_2\cdotp \hfill \end{array} $$

Consecutive reaction of the hydroxyl radical could take place either at the benzyl position of its isobutyl or ethyl substituents, on the tertiary carbon atom position of isobutyl side chain (group), or may occur directly at aromatic ring to give 1-(4-ethylphenyl)-2-methylpropan-1-ol, 1-(4-isobutylphenyl)ethanol, 1-(4-ethylphenyl)-2-methylpropan-2-ol, or 1-ethyl-4-isobutylphenol (IBP 2*a*, IBP 2*b*, IBP 2*c*, IBP 2*d*), respectively. Unfortunately, in our experiments, the exact structure of the products can not be confirmed by fragmentation ions but the photodegradation products IBP 2*a*, *b*, and *c* have been already identified in the literature [18, 26, 27, 29]. The subsequent degradation product IBP 3 of *m*/*z* value 159.1183 may correspond to 1-isobutyl-4-vinylbenzene, which is formed by elimination of formic acid (HCOOH group) from ibuprofen molecule or by dehydratation of product IBP 2b. This pathway has not yet been described in the literature.

Continued irradiation of the ibuprofen solution revealed formation of another degradation product (IBP 4, *m*/*z* value 221.1193). This compound was assumed to be the result of hydroxylation of the ibuprofen molecule still present in solution by OH^·^ radical. Hydroxylation may occur at the site of the molecule most susceptible to attack of the radical as well, i.e., at one of the two benzyl positions, at the tertiary carbon atom position or at the aromatic ring, as it was mentioned earlier. For that reason, four derivatives of IBP 4 molecule (IBP 4*a*, 4*b*, 4*c*, 4*d*) can be expected. All of these products have been already reported in the literature. 2-[4-(1-Hydroxy-2-methylpropyl)phenyl]propanoic acid (IBP 4*a*) and 2-hydroxy-2-[4-(2-methylpropyl)phenyl]propanoic acid (IBP 4*b*) have already been identified by Skoumal et al. [25] during the photoelectro-Fenton degradation and by Madhavan et al. [30] for sonophotocatalysis degradation, whereas product IBP 4*c* (2-[4-(2-hydroxy-2-methylpropyl)phenyl]propanoic acid) and IBP 4*d* (2-[hydroxy-4-(2-secbutyl)phenyl]propanoic acid) have been detected by Illes et al. [18] and by Mendez-Arriaga et al. [27] in heterogeneous TiO_2_ photocatalysis and in radiolysis. The final detected product IBP 5 has been formed already after 10 min of irradiation. Its concentration in examined solution continuously increased during exposition to Xe lamp light, whereas the concentration of ibuprofen simultaneously decreased. IBP 5 molecule with the *m*/*z* value of 273.1120 and fragmentation ions *m*/*z* 161.1336 and *m*/*z* 205.1242 characteristic for ibuprofen suggest that it may be the product of photodegradation of ibuprofen. Unfortunately, we are not able to predict its structure and the pathway. Ibuprofen dimer (*m*/*z* 411.2541) and its adduct with sodium (*m*/*z* 433.2360) were observed as well; however, these compounds were considered as products formed as a result of electrospray ionization rather than phototransformation. This is due to the fact that dimer and its adduct are observed since the beginning of the experiment and their concentration decreases proportionally to the decrease of IBP concentration.

**Fig. 3:**
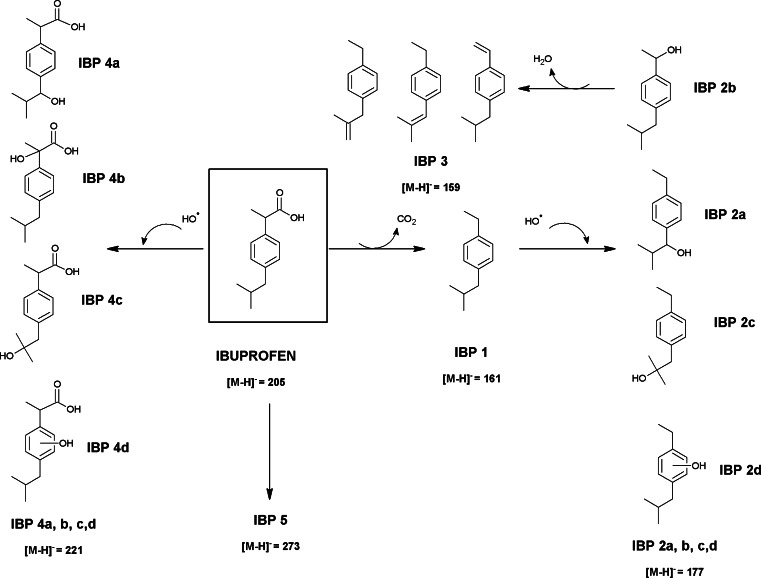
Suggested main transformation pathway of ibuprofen

Products detected by LC-QTOF-MS formed during the photocatalytic treatment and possible transformation pathways of ibuprofen in the aqueous environment are presented in Fig. 3.

#### Identification and confirmation of KET degradation products through laboratory degradation experiment

As it has been previously mentioned, ketoprofen had undergone photodegradation easier and with higher reaction rate than ibuprofen. The first degradation product (KET 1) has been identified after 10 s of the exposure of the ketoprofen solution to Xe lamp light. The structure corresponding to ion *m*/*z* 209.0972 may reveal decarboxylated ketoprofen molecule, what is often suggested in the literature as the first step of its degradation [31, 32]. The product KET 1 was completely degraded by photolytic irradiation within 80 s.

The compound KET 2 (3-(1-hydroxyethyl)benzophenone, *m*/*z* 225.0933) observed after 30-s lamp exposition is probably a product of hydroxylation at benzyl position of KET 1 molecule. This product is described in the literature [26] as a result of the vacuum ultraviolet photolysis. The signal from the by-product KET 1 was no longer registered in a sample taken after 80 s of irradiation, but the presence of the compound KET 2 was still recorded. The presence of the compounds KET 3, KET 4, KET 5, and KET 6 has also been revealed in the same sample. The most important fragment in the mass spectrum of product KET 3 is the ion of *m*/*z* 241.0883, which may correspond to the double hydroxylation of compound KET 1 or monohydroxylation of KET 2 at the aromatic ring. The exact position of the hydroxyl substituent at the aryl ring cannot be precisely established. Another detected compound KET 4 with the *m*/*z* value 197.0612 may represent the product after the cleavage of hydroxyethyl group from KET 3.

The appearance of compound KET 5 shows that the 3-(1-hydroxyethyl)benzophenone (product KET 2) is oxidized during irradiation and converted probably into 3-acetylbenzophenone with the *m*/*z* value 223.0771. The most important fragment in the mass spectrum of product KET 6 is the ion of *m*/*z* 239.0720, which may correspond to the elimination of acetyl group. Formation of all the above-mentioned products is confirmed by the fragmentation ions *m*/*z* 181.0670 and *m*/*z* 197.0613 which may correspond to unsubstituted benzophenone and hydroxybenzophenone, respectively. The products KET 3, KET 4, and KET 6 have not been yet reported in the literature.

Another detected product KET 7 has been formed after 30 s of irradiation. Its concentration in examined solution continuously increased during exposition to Xe lamp light, whereas concentration of ketoprofen simultaneously decreased. KET 7 product with the *m*/*z* value 221.1191 and fragmentation ion *m*/*z* 177.1300 suggest that it has been probably formed by oxidative ring opening of the ketoprofen molecule as was previously reported by Salgado et al. [9] for medium pressure UV photodegradation.

Irradiation of ketoprofen solution by the xenon lamp has been terminated after 5 min. No signals originating from ketoprofen and its degradation product KET 1 have been recorded then, whereas the presence of degradation products KET 2, KET 3, KET 4, KET 5, KET 6, and KET 7 was still detected in the examined solution.

**Fig. 4:**
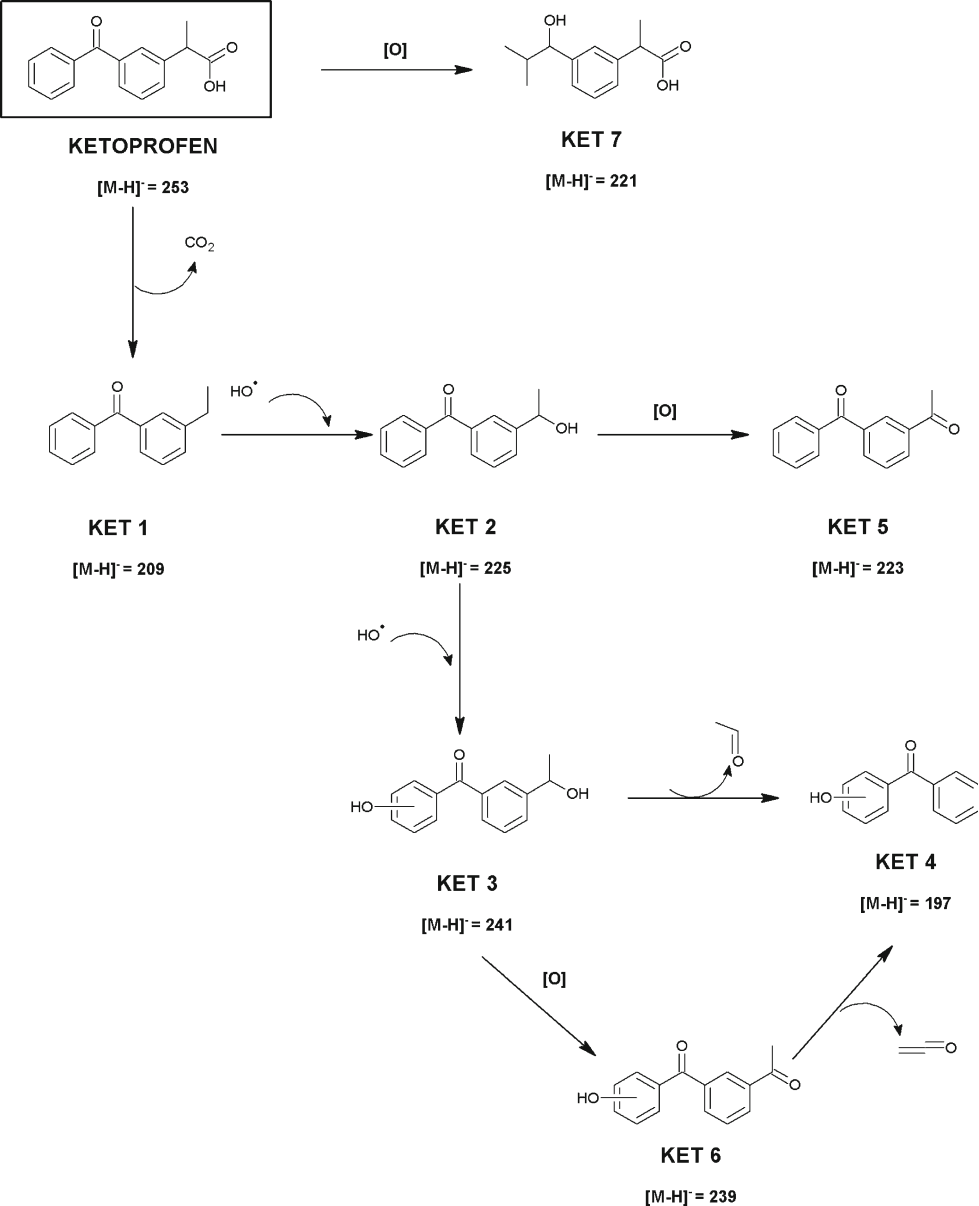
Suggested main transformation pathway of ketoprofen

On the basis of the degradation products identified in this experiment, the hypothetical ketoprofen degradation pathway has been suggested and schematically shown in Fig. 4.

#### Identification and confirmation of FUR degradation products through laboratory degradation experiment

**Fig. 5:**
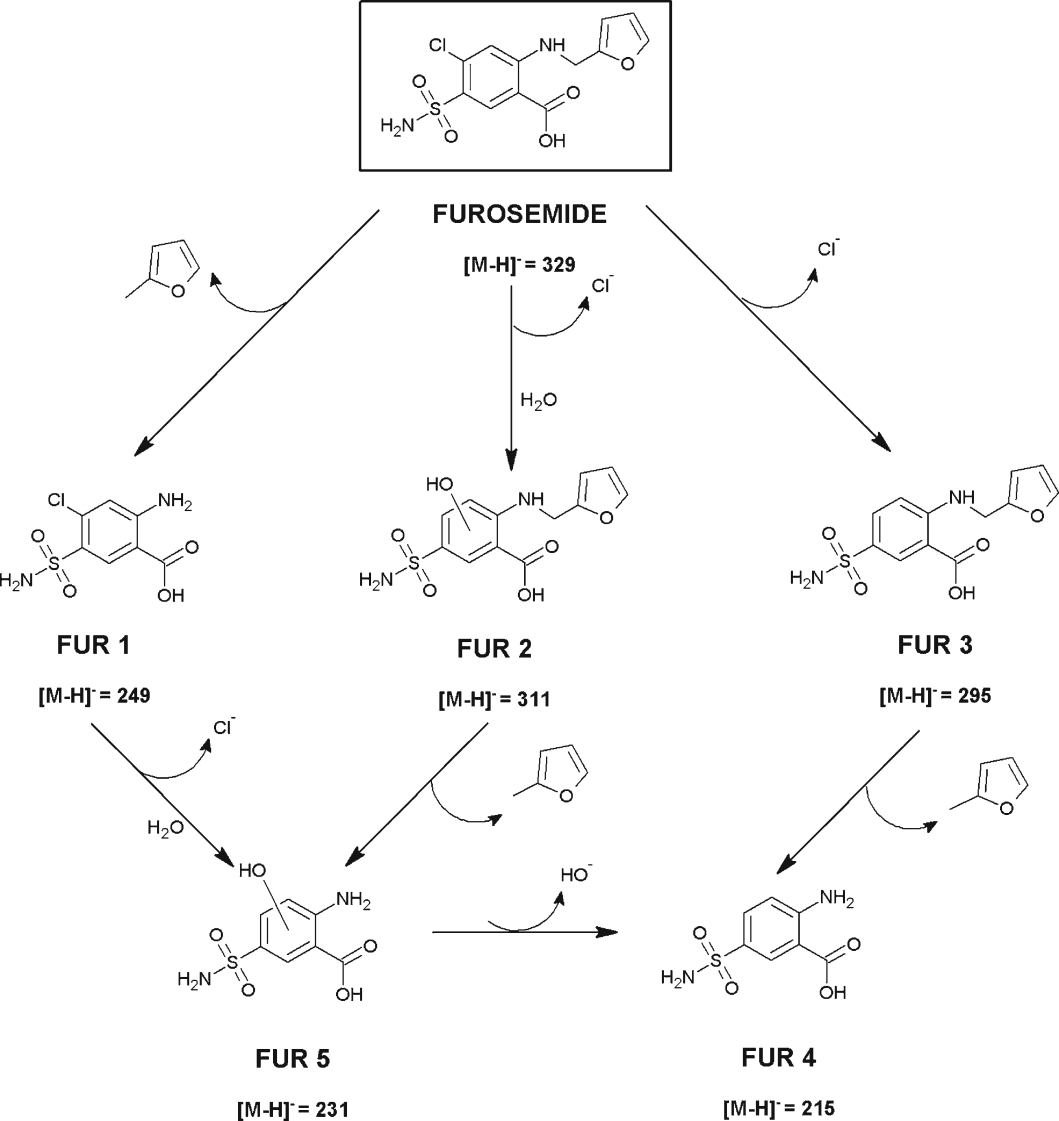
Suggested main transformation pathway of furosemide

The UV absorption spectra of furosemide overleap the Xe lamp emission spectrum; therefore, the photodegradation of this pharmaceutical occurs straightforward. The proposed photolysis pathway of furosemide is schematically presented in Fig. 5.

Product FUR 1 (*m*/*z* 248.9750) has been detected in a sample solution before irradiation. This product was probably obtained by cleavage of the furanylmethyl group due to the transformation processes occurring in real water sample. The signal from the other degradation products such as FUR 2, FUR 3, and FUR 5 has been recorded in a sample taken after 5 min of Xe lamp treatment. The most important fragment in the mass spectrum of product FUR 2 has the *m*/*z* 311.0327, which may correspond to the replacement of the chlorine atom by the hydroxyl group in the furosemide molecule. Product FUR 3 with the *m*/*z* value 295.0405 has been probably formed by dehalogenation of furosemide, whereas FUR 5 (*m*/*z* 231.0086) results by loss of furanylmethyl substituent and the replacement of the chlorine atom by the hydroxyl group. Formation of N-furfuryl-5-sulfamoylanthranilic acid (FUR 3) has been already reported by Vargas et al. [33] for the irradiation under oxygen atmosphere catalyzed by the presence of human serum albumin.

Compound FUR 4 which structure corresponds to the molecule of furosemide after the dehalogenation and elimination of the furanylmethyl group (*m*/*z* 215.0140) has been identified in the sample taken after 15 min of continuous exposure of the solution. Furosemide solution was irradiated with a xenon lamp light continuously for 1 h, that is, to the point where furosemide molecule was completely decomposed, but identification of its degradation products was still possible.

The immediate formation of product FUR 1 evidences that cleavage of the furanylmethyl group from furosemide molecule occurs easily. Further degradation pathways are difficult to anticipate. FUR 5 may be formed as the result of replacement of the chlorine atom by the hydroxyl group. However, the furosemide molecule may first be substituted by hydroxyl group giving compound FUR 2, and subsequently, it may be deprived of the furanylmethyl group (FUR 5). The compound FUR 5 can undergo further reaction during exposure to radiation (cleavage of hydroxyl group) and in a result may give end product FUR 4. Another way leading to FUR 4 may be dehalogenation of furosemide to FUR 3, followed by lose of a furanylmethyl group (FUR 4).

### Dynamics of the transformation products generated during the irradiation

**Fig. 6:**
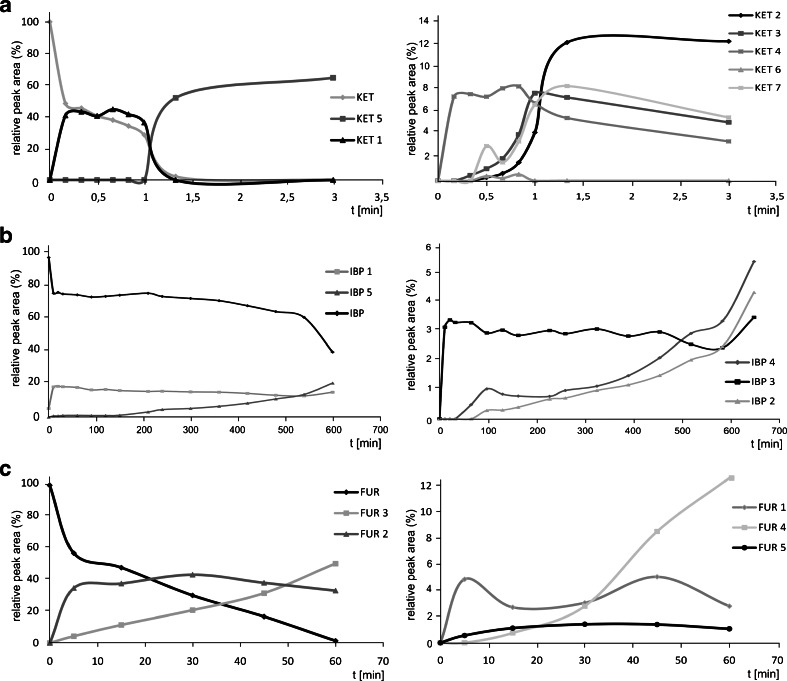
Relative peak area of the PhACs and corresponding TPs in the photodegradation experiment performed in river water **a** for ketoprofen, **b** ibuprofen, and **c** furosemide

The irradiation of a sample containing ibuprofen led to the formation of the main TP product IBP 1 (Fig. 6a) which was observed in the river water before the exposure to light suggesting its creation within transformation processes such as hydrolysis rather than photodegradation. The further degradation was observed by the proportional decrease of the amount of IBP and IBP 1 to the increase of the level of IBP 2 and 4. As it can be seen in the degradation pathway scheme (Fig. 3), the transformation of either IBP to IBP 4 or IBP 1 to 2 was probable to occur. IBP 3 was the most persistent and stable compound that occurs in the sample right after the beginning of the irradiation and did not degrade at any amount within the experiment. Although it was not possible to elucidate the IBP 5 structure, it was considered as one of TPs due to the increase of its amount while performing the experiment while IBP was degrading.

Photolysis of ketoprofen generated three compounds appearing in the highest amount, KET 1, 2, and 5 (Fig. 6b). The amount of KET 1 was increasing proportionally to the decrease of the concentration of ketoprofen; however, both compounds degraded below detection limit after the first minute of irradiation. The lack of KET in the solution was not consistent with the further lack of degradation products which were observed until 5 min exposure to Xe lamp light. The transformation of KET 1 to KET 2 and later to KET 5 was observed after 1 min exposure, meaning after the degradation of KET 1. The formation of two other TPs (KET 3 and 4) was observed as well. As long as the amount of KET 3 increased slowly along irradiation time until 1 min, the content of KET 4 was observed since the beginning of the light exposure lasting at a similar level for 3 min, and thus, this analyte was considered as a persistent compound. A very small amount of KET 6 was noted; however, it was considered as an intermediate degradation product which was formed from KET 3 and directly transformed to KET 4. Additional TP was found, KET 7, formed through the decay of the aromatic ring, which level was increasing along the irradiation time. It was consistent with degradation pathway elucidated for ketoprofen (Fig. 4).

The phototransformation of furosemide in photodegradation experiment in river water resulted in two main TPs (FUR 2 and 3, Fig. 6c); however, other products FUR 1, 4, and 5 were identified at very low peak area close to LOD (*S*/*N* = 3). Compounds FUR 2 and 5 were considered as the most persistent ones since their concentration level increased within first few minutes and was stable during further irradiation. However, when the level of FUR 2 and 5 finally started to decrease, it was observed that the amount of the product FUR 4 increased rapidly since it was created as a result of the transformation of the two previous TPs. These conclusions are consistent with the transformation pathway of furosemide (Fig. 5).

The experiment proved the formation of many TPs of ibuprofen, ketoprofen, or furosemide; some of them appeared persistent and were reported here for the first time. It leads to the conclusion that tracing their occurrence in the aqueous environment as well as in WWTPs is of a great importance and necessary to perform. Future research on the ecotoxicity potential of identified compounds should be conducted.

### The occurrence of the selected pharmaceuticals and TPs in aqueous environment

The presence of parent compounds and their identified transformation products was determined in the waste water (WW) influent and effluent, treated and untreated water from WTP, and Radunia River exposed to anthropogenic activity. Screening of all compounds was performed in target MS/MS mode for all samples using the window tolerance of 5 ppm. The confirmation of selected pharmaceuticals and TPs in environmental waters samples was accomplished by the structural information of deprotonated parent compound obtained from the fragmentation experiment provided by LC-QTOF-MS/MS analysis. To avoid false-positive findings, identification of the compounds was based on LC retention time of the analyte with the deviation <2 %, accurate mass measurements of the parent ion and accurate mass measurement of at least one specific product ion together with their relative abundance with a mass error below 5 ppm, comparing with the data obtained during laboratory degradation experiment (Table 2). Unfortunately, some accurate mass measurements of parent and product ions gave mass errors higher than 5 ppm. It was acceptable as long as MS/MS spectra were similar to those obtained during laboratory photodegradation. However, it was found that in some samples, especially WW influent, the MS/MS spectra were unclear and some of the specific product ions were not observed principally due to the masking effect of highly abundant isobaric interferences. In such cases, it was not possible to state unambiguously about the presence of the compound, and thus, such results were rejected from further consideration.

Data obtained for selected pharmaceuticals and TPs on their occurrence in various water samples are presented in Table 3. Only ketoprofen was detected in untransformed form in considered waters. Most of the TPs of selected drugs were found at least once in all aqueous samples. However, FUR 5, IBP 3, and KET 4 and 5 were not detected in any sample. WW influent was the most contaminated by TPs derived from all three pharmaceuticals. Considering ketoprofen, its products KET 2 and 3 were determined most often in water samples (18/25 samples). Surprisingly, KET 6, which was considered as an intermediate product, was found in WW influent (5/5 samples) and effluent (2/5 samples). In the case of ibuprofen, IBP 1 was found in almost all water samples; however, its presence may be caused by transformation processes occurring in natural waters (e.g., hydrolysis or (less probable) biodegradation by the microorganisms present in the matrix) rather than as a result of photolysis. TPs of furosemide were noticed rarely; however, FUR 1 and 4 were found in treated water (3/5 samples) what may indicate on their formation during water treatment process.

**Table 3:** The occurrence of selected pharmaceuticals and their TPs in various water samples

Compound	Positive samples/*n* samples				
	WW influent	WW effluent	Radunia river	Untreated water	Treated water
FUR	0/5	0/5	0/5	0/5	0/5
FUR 1	2/5	0/5	1/5	2/5	3/5
FUR 2	1/5	1/5	1/5	2/5	0/5
FUR 3	0/5	0/5	1/5	0/5	0/5
FUR 4	0/5	0/5	0/5	0/5	3/5
FUR 5	0/5	0/5	0/5	0/5	0/5
IBP	0/5	0/5	0/5	0/5	0/5
IBP 1	5/5	4/5	4/5	1/5	4/5
IBP 2	2/5	2/5	0/5	2/5	1/5
IBP 3	0/5	0/5	0/5	0/5	0/5
IBP 4	2/5	3/5	1/5	1/5	2/5
IBP 5	5/5	0/5	0/5	1/5	2/5
KET	5/5	3/5	5/5	4/5	4/5
KET 1	1/5	2/5	0/5	3/5	2/5
KET 2	1/5	3/5	4/5	5/5	5/5
KET 3	5/5	4/5	5/5	2/5	1/5
KET 4	0/5	0/5	0/5	0/5	0/5
KET 5	0/5	0/5	0/5	0/5	0/5
KET 6	5/5	2/5	1/5	0/5	0/5
KET 7	1/5	1/5	3/5	3/5	0/5

In order to assess the degree of the elimination of pollutants from WWTPs, it would be necessary to determine the presence of pharmaceuticals and TPs in the sewage sludge, which particles can adsorb impurities. In such case, they will not be present in the effluent stream; however, these compounds can infiltrate from disposed sludge to ground water leading to its contamination. In some cases, only TPs were detected, so the determination of transformed compounds in the aqueous environment is justified. Contaminants can spread between ground and surface water, which are the source of drinking water, and thus, the presence of pharmaceuticals and their TPs in various water samples should be monitored.

The studies confirm that the control of the presence of contaminants should include the knowledge of their behavior (fate) in the aquatic environment. Absence of pharmaceuticals in the form in which they are released to the environment does not mean that they are not present in a transformed form. The conducted experiment shows that furosemide and ibuprofen were not present in the original form, but its TPs have been identified. It is possible that the same photodegradation products could occur also as a result of biodegradation.

## Conclusion

LC-QTOF-MS is a very powerful tool for the identification of TPs of pharmaceuticals in the aqueous environment. Five degradation products of IBP photolysis, seven products of KET, and five of FUR were identified using MS/MS experiment with accurate mass measurements followed by the creation of transformation pathway for each compound. To the best of our knowledge, some of the identified compounds along with its transformation pathway are presented for the first time. Of major significance is the fact that these compounds are repeatedly detected in the aqueous environment: WW influent and effluent, river water, and untreated/treated water. Actual data showed that although the original compound is not present as such (IBP and FUR), their TPs are still present what leads to the conclusion that wastewater treatment processes are not efficient, and the impact on the receiving waters is unfortunately unknown. Further studies will be focused on monitoring of the environment and assessment of their ecotoxicity.

## Electronic supplementary material

Below is the link to the electronic supplementary material.

ESM 1 (PDF 5,107 kb)
