# Supplementary material for: Elucidation of transformation pathway of ketoprofen, ibuprofen, and furosemide in surface water and their occurrence in the aqueous environment using UHPLC-QTOF-MS
Source: Anal Bioanal Chem. 2014 Jan 23;406(15):3667–80. doi: 10.1007/s00216-014-7614-1 (PMC4026630; doi:10.1007/s00216-014-7614-1)
Supplement: Supplementary file 1 — (PDF 5,107 kb) [file 216_2014_7614_MOESM1_ESM.pdf]

Analytical and Bioanalytical Chemistry

Electronic Supplementary Material

Elucidation of transformation pathway of ketoprofen, ibuprofen, and furosemide in surface water and their occurrence in the aqueous environment using UHPLC-QTOF-MS

A. Jakimska, M. Śliwka-Kaszyńska, J.Reszczyńska, J. Namieśnik, A. Kot-Wasik

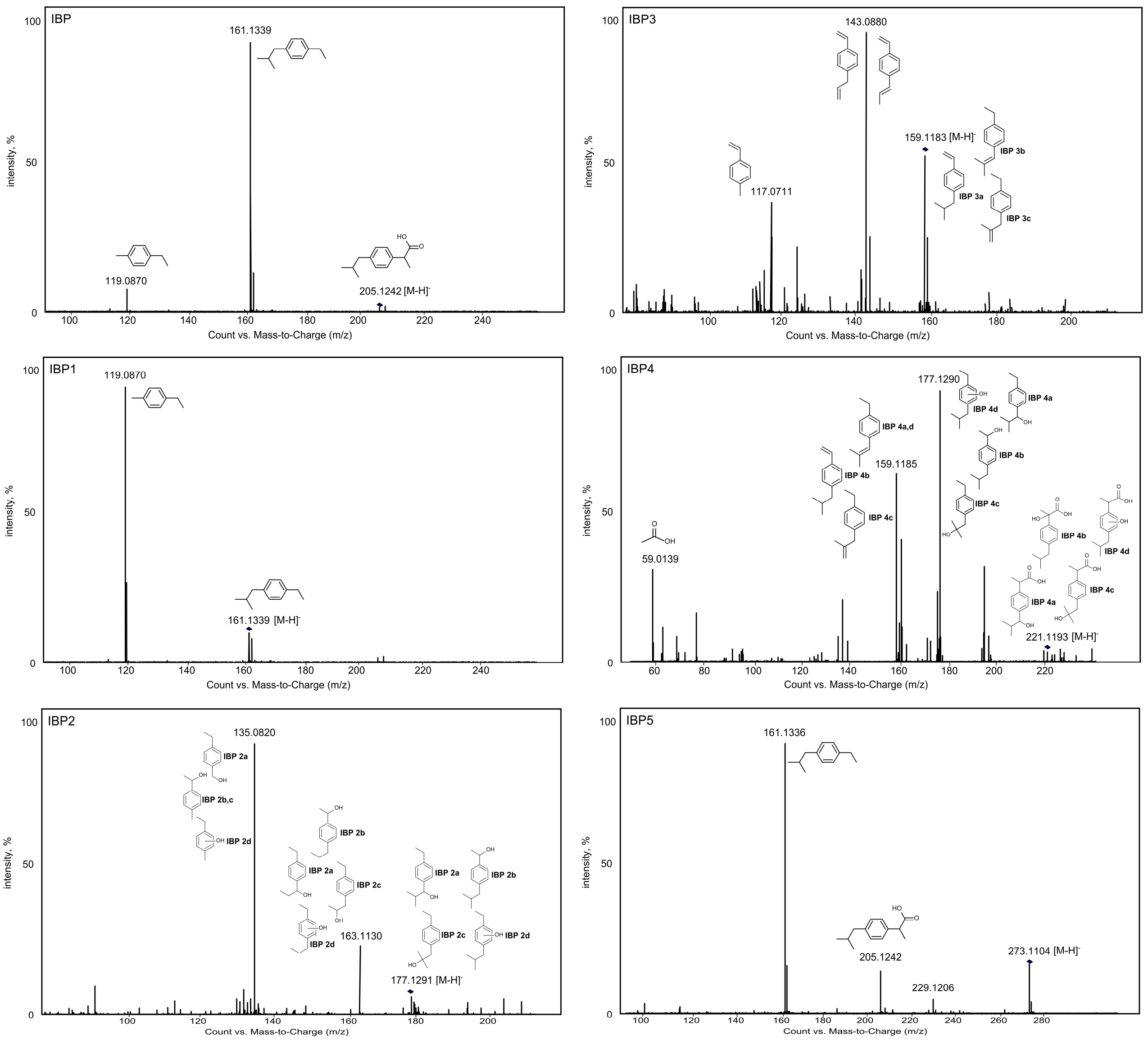

Fig. S1. Mass spectra obtained for ibuprofen and its TPs during the photodegradation experiment

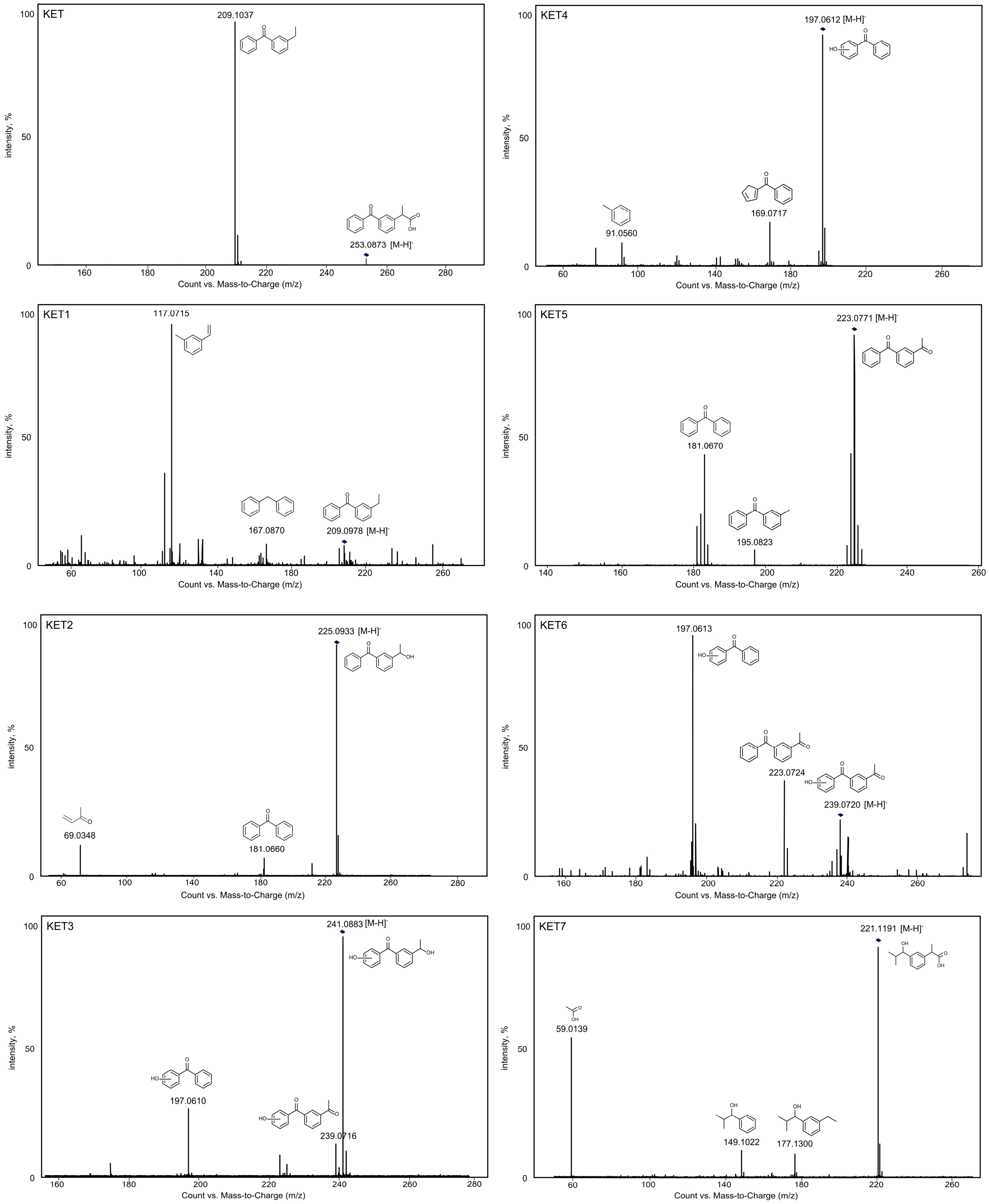

Fig. S2. Mass spectra obtained for ketoprofen and its TPs during the photodegradation experiment

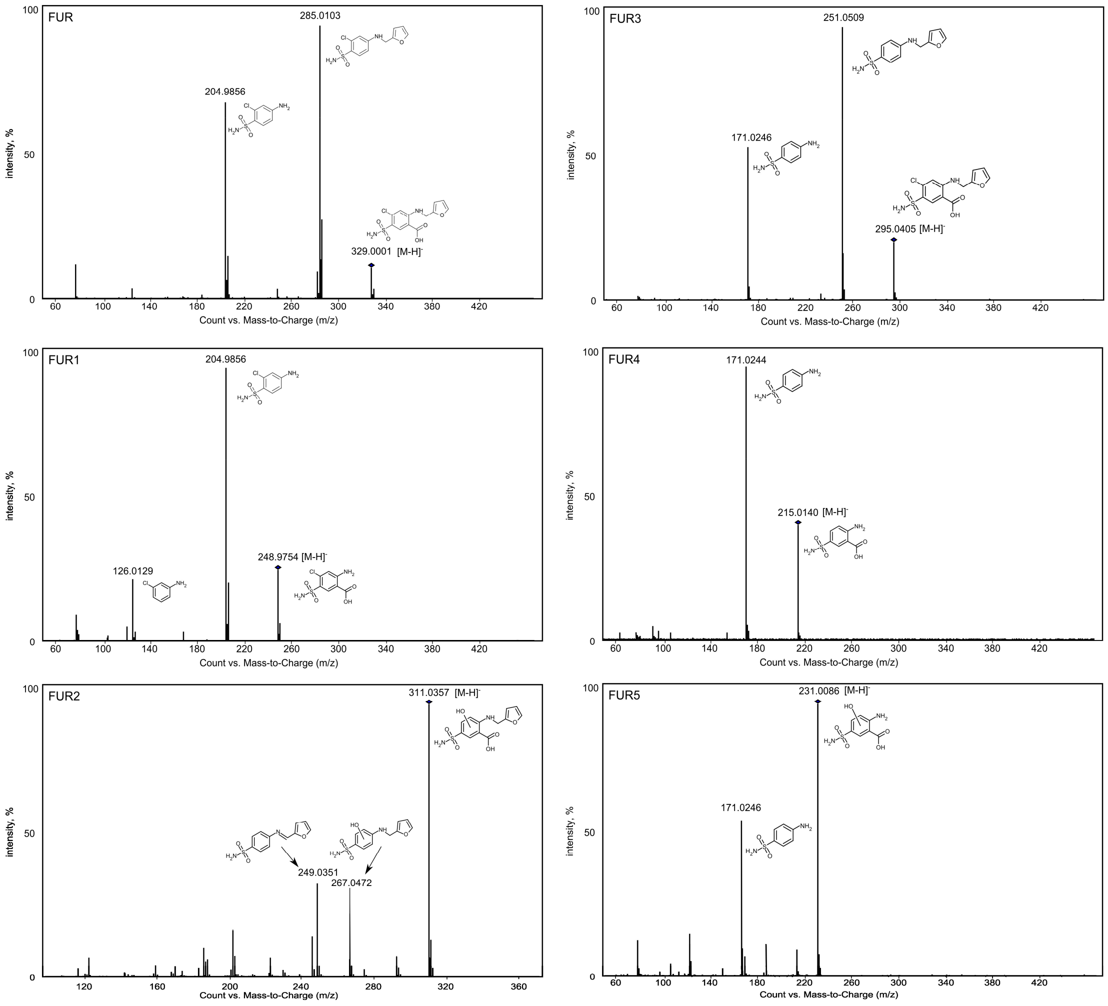

Fig. S3. Mass spectra obtained for furosemide and its TPs during the photodegradation experiment
